# Nanoscale Organisation of Ryanodine Receptors and Junctophilin-2 in the Failing Human Heart

**DOI:** 10.3389/fphys.2021.724372

**Published:** 2021-10-08

**Authors:** Yufeng Hou, Jizhong Bai, Xin Shen, Oscar de Langen, Amy Li, Sean Lal, Cristobal G. dos Remedios, David Baddeley, Peter N. Ruygrok, Christian Soeller, David J. Crossman

**Affiliations:** ^1^Department of Physiology, University of Auckland, Auckland, New Zealand; ^2^Institute for Experimental Medical Research, Oslo University Hospital, University of Oslo, Oslo, Norway; ^3^Department of Pharmacy and Biomedical Science, Health and Engineering, La Trobe University, Bendigo, VIC, Australia; ^4^Faculty of Medicine and Science, University of Sydney, Sydney, NSW, Australia; ^5^Victor Chang Cardiac Research Institute, Darlinghurst, Sydney, NSW, Australia; ^6^Auckland Bioengineering Institute, University of Auckland, Auckland, New Zealand; ^7^Department of Cardiology, Auckland City Hospital, Auckland, New Zealand; ^8^Biomedical Physics, University of Exeter, Exeter, United Kingdom

**Keywords:** t-tubules, ryanodine receptor, collagen VI, fibrosis, junctophilin

## Abstract

The disrupted organisation of the ryanodine receptors (RyR) and junctophilin (JPH) is thought to underpin the transverse tubule (t-tubule) remodelling in a failing heart. Here, we assessed the nanoscale organisation of these two key proteins in the failing human heart. Recently, an advanced feature of the t-tubule remodelling identified large flattened t-tubules called t-sheets, that were several microns wide. Previously, we reported that in the failing heart, the dilated t-tubules up to ~1 μm wide had increased collagen, and we hypothesised that the t-sheets would also be associated with collagen deposits. Direct stochastic optical reconstruction microscopy (dSTORM), confocal microscopy, and western blotting were used to evaluate the cellular distribution of excitation-contraction structures in the cardiac myocytes from patients with idiopathic dilated cardiomyopathy (IDCM) compared to myocytes from the non-failing (NF) human heart. The dSTORM imaging of RyR and JPH found no difference in the colocalisation between IDCM and NF myocytes, but there was a higher colocalisation at the t-tubule and sarcolemma compared to the corbular regions. Western blots revealed no change in the JPH expression but did identify a ~50% downregulation of RyR (*p* = 0.02). The dSTORM imaging revealed a trend for the smaller t-tubular RyR clusters (~24%) and reduced the t-tubular RyR cluster density (~35%) that resulted in a 50% reduction of t-tubular RyR tetramers in the IDCM myocytes (*p* < 0.01). Confocal microscopy identified the t-sheets in all the IDCM hearts examined and found that they are associated with the reticular collagen fibres within the lumen. However, the size and density of the RyR clusters were similar in the myocyte regions associated with t-sheets and t-tubules. T-tubule remodelling is associated with a reduced RyR expression that may contribute to the reduced excitation-contraction coupling in the failing human heart.

## Introduction

The highly ordered nature of the excitation-contraction coupling (ECC) machinery of ventricular myocytes is recognised as the key cellular structure enabling a tightly regulated and synchronised contraction (Bers, 2002). This includes the extraordinarily complex invaginations of the ventricular myocyte sarcolemma, namely, the transverse tubules (t-tubules), although this term is not always correct as axial tubules are also present (Soeller and Cannell, 1999). The t-tubules facilitate the rapid conduction of action potential to the cell interior, facilitating a nearly simultaneous calcium ion (Ca^2+^) release, and hence, contractions across the cell volume (Crocini et al., 2014). The initiation of the cell-wide Ca^2+^ transient occurs at specialised nanostructures called the cardiac junction or dyadic cleft, which lies in close apposition of the sarcolemma with the sarcoplasmic reticulum (Page and Surdyk-Droske, 1979). Upon depolarisation, the L-type Ca^2+^ channels located on the sarcolemma side of the dyad open, triggering the Ca^2+^ to enter the cell. The gap between the sarcolemma and the sarcoplasmic reticulum (SR) at the dyad is ~10 nm and provides a restricted volume that concentrates the incoming Ca^2+^ to the levels required to trigger the opening of the SR Ca^2+^ released from the ryanodine receptor (RyR)-2, thereby, initiating a cell-wide Ca^2+^ release (Takeshima et al., 2000).

It is now well-recognised that the pathological remodelling of the ECC machinery, particularly the loss of t-tubules, is a major driver of the loss of contractile function in the failing heart. Several studies have demonstrated the loss of the t-tubules in heart failure (HF), including studies on animal models (Louch et al., 2006; Song et al., 2006; Heinzel et al., 2008) and humans (Heling et al., 2000; Crossman et al., 2011; Zhang et al., 2013; Guo et al., 2015; Wang et al., 2018). Furthermore, physiological studies have demonstrated that the loss of t-tubules is linked to the loss of cardiac function. Isolated myocyte studies have confirmed that cells lacking t-tubules, either from failing hearts or through experimental detubulation, have reduced and dyssynchronous Ca^2+^ release (Kawai et al., 1999; Louch et al., 2006; Song et al., 2006). Live-cell imaging of whole rat hearts demonstrated that t-tubule remodelling is correlated with cardiac function and precedes the development of HF, implicating its causative role in HF (Wei et al., 2010). In findings, it is consistent that the t-tubule remodelling in HF is associated with a loss of transverse components and an increase in axial components (Song et al., 2006). For example, we have reported that contractile function within the failing human heart is strongly associated with the number of transverse components (Crossman et al., 2015c). This would result in the loss of dyads or initiations sites required to trigger Ca^2+^ transients, as the transverse components of the t-tubules are aligned with the Z discs where the majority of the sarcoplasmic reticulum Ca^2+^ release channels and the RyR are located. Notably, a quantitative electron microscopy study has demonstrated that the loss of t-tubules is also associated with the loss of dyads in the failing human heart (Zhang et al., 2013).

The protein junctophilin (JPH)-2 is proposed as a key driver in the t-tubule remodelling in HF (Beavers et al., 2014). It forms a physical link between the plasma membrane and the RyR on the SR and is critical for the formation of dyads. The knockout of this protein is embryonically lethal, demonstrating that it is fundamental for life (Takeshima et al., 2000). Furthermore, cardiac-specific knockdown in adult mice results in the loss of t-tubule, impaired calcium handling, and the development of HF (Van-Oort et al., 2011). Super-resolution imaging of these animals revealed a loss of JPH co-localisation with the RyR (Munro et al., 2016). The JPH down-regulation was also found in the hypertrophic and dilated mouse models of HF (Minamisawa et al., 2004). In the rat thoracic aortic banding model of HF, there was a loss of t-tubules which is correlated with the drop in JPH expression (Wei et al., 2010). However, these results may be model dependent as JPH expression is not linked to t-tubule remodelling in the sheep and ferret models of HF (Caldwell et al., 2014). Nevertheless, reduced levels of JPH have been reported in human hypertrophic cardiomyopathy, dilated cardiomyopathy and ischaemic cardiomyopathy suggesting its important role in human HF (Landstrom et al., 2007; Zhang et al., 2013; Guo et al., 2015; Xiao et al., 2018). However, it is not known if the loss of co-localisation between JPH and RyR occurs in the failing human heart. This would be expected if the JPH down-regulation were driving the t-tubule remodelling.

Ryanodine receptors are organised into multiunit clusters within the cardiac myocytes which are responsible for producing Ca^2+^ sparks, which constitute the fundamental unit of Ca^2+^ release from the SR (Cheng et al., 1993). RyR clustering was first observed in electron microscopy studies where the giant tetrameric RyR molecule (~2 MDa) was attributed to electron-dense particles packing the cardiac junctions with an approximate centre to centre spacing of 29 nm (Franzini-Armstrong et al., 1999). Epifluorescence and confocal microscopy of the immune-labelled cells and tissue have confirmed the organisation of RyR into rows of discrete puncta, aligned with the Z discs (Soeller et al., 2007). The co-labelling of the t-tubules revealed that many of the RyR puncta were not part of the cardiac junction (Jayasinghe et al., 2012b) and Ca^2+^ release from these clusters was thought to be initiated by Ca^2+^ released from neighbouring clusters (Soeller et al., 2007). However, it was not possible to acquire a detailed image of the RyR cluster organisation in diffraction-limited imaging due to the resolution limit of ~250 nm. New insight was provided by the advent of super-resolution microscopy with a ~10-fold improvement in the resolution which demonstrated that RyR cluster sizes in rat myocytes followed an exponential distribution dominated by very small clusters, many of which were not detected by the diffraction-limited imaging (Baddeley et al., 2009). Many of these small clusters were within <100 nm of the neighbouring clusters and were proposed to act functionally as a single calcium release unit (CRU) or supercluster (Baddeley et al., 2009).

A super-resolution imaging of atrial myocytes in sheep with persistent atrial fibrillation (AF) demonstrated that the RyR clusters were more densely crowded. The CRUs were larger and contained increased individual clusters, although the average cluster size was similar to control animals. This geometric arrangement was associated with a higher spark frequency, increased numbers of larger sparks, and slowed kinetics that would contribute to increased Ca^2+^ release in AF (Macquaide et al., 2015). Recently, a super-resolution imaging of rat ventricle myocytes with ischaemic HF identified smaller RyR clusters but organised into geometrically larger calcium release units containing more clusters but fewer RyRs (Kolstad et al., 2018). The increased fraction of smaller clusters were linked to a rise in the non-spark “silent” Ca^2+^ leaks. The larger dispersed CRUs were associated with an increased spark mass with slowed kinetics and desynchronised total Ca^2+^ transient (Kolstad et al., 2018). In a third study, expansion microscopy was used to examine the isolated right ventricular myocytes of a rat model with pulmonary hypertension that causes right-sided HF. This study revealed RyR cluster fragmentation as well as a non-uniform hyper-phosphorylation within the cluster centre with a diminished ability to initiate Ca^2+^ release in simulations (Sheard et al., 2019).

These data support the premise that RyR cluster organisation contributes to the kinetics of Ca^2+^ release within the myocyte. In contrast to these detailed animal model-based studies, the nanoscale organisation of RyR in human cardiac myocytes and the impact that disease may have on this organisation are currently unknown.

Recently, a 3D confocal microscopy study of the failing human heart identified a new previously unappreciated feature of t-tubule remodelling, namely, large flattened sheet-like structures called t-sheets. Their presence was associated with larger RyR-to-sarcolemma distances and delayed Ca^2+^ transient (Seidel et al., 2017a) compared with patients with fewer t-sheet structures. The authors suggested 2D imaging, which was used in previous studies and resulted in t-sheets mistakenly identified as axial tubules. In a different study of t-tubule changes in human hearts, we reported that dilated t-tubules in the failing human heart contain increased collagen, suggesting that fibrosis could be a contributing factor driving t-tubule remodelling (Crossman et al., 2017). We suspected that t-sheets, like dilated t-tubules, would also contain collagen. This proposition is supported by a previous study that used wheat germ agglutinin (WGA) labelling to visualise t-sheets (Seidel et al., 2017a). We identified that a major component of WGA labelling in the failing human heart was collagen VI (Crossman et al., 2017), an observation that indicates that t-sheets likely contain collagen.

The work presented here has three objectives: (1) To determine if reduced co-localisation of JPH and RyR is correlated with the loss of t-tubules and cardiac junctions previously documented in the failing human heart; (2) Determine if there are changes in RyR cluster organisation in human HF that could contribute to disrupted Ca^2+^ release in human HF; (3) Determine if t-sheets were associated with luminal collagen deposition and changes in RyR cluster organisation. To achieve these objectives we used a combination of confocal and super-resolution microscopy coupled with western blots to examine non-failing (NF) donor hearts and the hearts of patients with idiopathic dilated cardiomyopathy (IDCM).

## Methods

### Human Cardiac Tissue

Idiopathic dilated cardiomyopathy tissue samples were obtained from the Auckland City Hospital, New Zealand. The NF human tissue was supplied by the Organ Donation New Zealand, and from the University of Sydney in collaboration with St Vincent's Hospital, Sydney. The human tissue used in this study was collected with the informed and written consent of transplant patients or from the families of the organ donors of NF hearts following the principles in the Declaration of Helsinki. Ethical approval was provided by the Health and Disability Ethics Committees in New Zealand (NTY/05/08/050/AM05), Human Research Ethics Committees at the University of Sydney (2016/7326), and St Vincent's Hospital (H03/118). The tissue samples were preserved as previously described (Crossman et al., 2017). The patient details are presented in Table 1.

**Table 1:** Donor and patient characteristics.

**Donor**			**IDCM**					
**Age, y**	**Sex**	**Code**	**Age, y**	**Sex**	**NYHA**	**LVEF, %**	**LVEDD, mm**	**LVESD, mm**
62	F		57	F	III	15	72	65
57	F		54	M	III–IV	18	80	74
54	F		52	M	III–IV	17	67	61
48	F	5.089	18	M	IV	15	68	65
53	M	4.083	49	F	III	21	74	64
44	F	5.090	48	F	III–IV	18	69	65
19	M	7.012	54	M	IV	~35%	48	NA
			58	M	II–IV	9	82	NA
			21	M	III–IV	19	76	68
			60	M	I on LVAD	25	76	65
			17	F	III–IV	20	98	82
			21	M	III	14	90	82

*NYHA, New York Heart Association classification; LVAD, left ventricle assist device; LVEF, left ventricle ejection fraction; LVEDD, left ventricle end-diastolic diameter; LVESD, left ventricle end-systolic diameter; Code, Sydney Heart Bank de-identified sample code*.

### Immunohistochemistry

For the direct stochastic optical reconstruction microscopy (dSTORM) imaging, sections with 10 μm thickness were cut from the frozen tissue blocks and mounted on coverslips coated with poly-d-lysine. The sections were then permeabilized with 1% Triton X100 in phosphate buffered saline (PBS) for 15 min, washed in PBS followed by incubation with FX signal enhancer (ThermoFisher) for 1 h. For JPH, RyR, and WGA labelling, the sections were incubated overnight at 4°C with polyclonal rabbit anti-JPH [1:100, custom made (Van-Oort et al., 2011)] and mouse monoclonal anti-RyR (1:100, MA3-916, ThermoFisher). The sections were washed and incubated with goat anti-rabbit Alexa Fluor 680 (1:100, ThermoFisher), rabbit anti-mouse Alexa Fluor 750 (1:100, ThermoFisher) and WGA Alexa Fluor 594 (1:200, ThermoFisher). For the RyR and WGA labelling, the sections were incubated similarly with anti-RyR (1:100, MA3-916, ThermoFisher), followed by goat anti-mouse Alexa Fluor 680 (1:100, ThermoFisher) and WGA Alexa Fluor 488 (1:100, ThermoFisher). For confocal imaging, the same general labelling procedure was followed. For Col-I, WGA, and RyR labelling, the sections were first incubated with rabbit anti-Col I (ab292, Abcam) and anti-RyR (1:100, MA3-916, ThermoFisher) followed by goat anti-rabbit Alexa Fluor 488 (1:100, ThermoFisher), goat anti-mouse Alexa Fluor 647 (1:100, ThermoFisher), and WGA Alexa Fluor 594 (1:200 ThermoFisher). For Col-VI and RyR labelling, the sections were first incubated with rabbit-anti Col-VI (1:100, ab6588, Abcam) and anti-RyR (1:100, MA3-916, ThermoFisher) followed by goat anti-rabbit Alexa Fluor 488 (1:200, ThermoFisher) and goat anti-mouse Alexa Fluor 647 (1:200, ThermoFisher).

### Confocal Imaging

The samples prepared for confocal imaging were mounted in 90% glycerol in PBS. The samples previously used for super-resolution imaging were imaged in the switching buffer as described below. Confocal images were obtained on a Zeiss LSM 710 inverted confocal microscope. 3D image stacks were collected with a 63 × NA 1.4 oil-immersion objective (94 × 94 pixel spacing, 250 nm Z-slicing). The image stacks were then deconvolved with the Richardson-Lucy algorithm as previously described (Soeller and Cannell, 1999). The volume rendering of t-sheets was performed with the 3D Viewer in the Fiji distribution of ImageJ and Paraview.

### Correlative Confocal and dSTORM Super-Resolution Imaging

The correlative confocal and super-resolution imaging was performed as previously described (Soeller et al., 2017). Briefly, the tissue sections were mounted with an imaging buffer containing 90% glycerol and 10 mM cysteamine (MEA) in PBS an hour before imaging. The dSTORM images were collected on a Nikon TE200 inverted total internal reflection microscope modified for dual colour localisation microscopy. This involved focusing a solid-state 671 nm laser through a 60x NA 1.49 oil-immersion TIRF objective (Nikon) as a highly inclined light sheet to achieve a ~10^9^ W/m^2^ non-TIRF illumination within a 20 μm-wide area up to several microns deep within the sample. The emitted light was then passed through a dichroic mirror (Q680LP, Chroma Technology) and an emission filter (XF3104-690ALP, Omega optical) before being split into two spectral channels using a custom-built splitter device built as previously described (Baddeley et al., 2011). This provided a 2D axial super-resolution imaging with a localisation precision of ~15 nm as previously reported (Baddeley et al., 2011). There were numerous single RyR with a ~30 nm full width half maximum. Fourier ring correlation indicated a resolution of ~60 nm. The axial resolution of dSTORM was diffraction limited at ~700 nm. The actively switching transversely orientated myocytes were imaged for 20,000–40,000 frames at a rate of 50 ms per frame. The light emitting from the sample was captured on two halves of the cooled EM-CCD chip of an IXon DV887DCS-BV camera (Andor Technology, Belfast). After a super-resolution imaging, correlative confocal Z-stacks of WGA labelling was captured to provide imaging of the t-tubules and cell border (see confocal imaging section for microscope details).

### dSTORM Image Analysis

Custom written algorithms coded in Python were used to identify single-molecule events and determine fluorophore designation by ratio-metric analysis of camera channels (Baddeley et al., 2011). The localisations points were then rendered using jittered triangulation onto 5 nm × 5 nm pixel spacing 2D TIFF image. The colocalisation of RyR and JPH was determined using the method previously described (Jayasinghe et al., 2012a) with the addition of separating the labelling of RyR and JPH into the t-tubular, sarcolemma, and corbular regions based on a binary mask created from the correlative confocal image of WGA (Crossman et al., 2015a). The term “corbular” refers to the regions of the SR without cardiac junctions, alternatively called non-junctional SR. The RyR cluster size was calculated from the rendered images thresholded using the PYME signal fraction method set to 50%. The cluster sizes were then measured *via* a custom python code utilising the scipy ndimages library. A filter was applied to the measured sizes where all clusters areas below 1 RyR were excluded from the analysis. For the cluster edge to edge nearest neighbour distances, each cluster was iterated over with a Euclidean distance transform (EDT). A cluster with the lowest transform range from the selected cluster was determined to be the nearest neighbour. The superclusters were identified through an EDT of the RyR mask inverse and all pixels with the value < 50 nm were selected. The cluster analyses were separated into the t-tubular, sarcolemma and corbular regions. The cluster and tetramer densities were expressed per cell area and rather than in the area of the t-tubular, sarcolemma and corbular masks. These structures were below the resolution limit and could not be accurately estimated for normalisation. The JPH event counts associated with each RyR cluster were measured using the regions of interest obtained from RyR cluster masks to measure events in the corresponding JPH channel. Each JPH image was normalised to the mean event count.

### Western Blotting

Tissue samples were homogenised in urea/thiourea extraction buffer (87% glycerol, 7 M Urea, 2 M thiourea, 15 mM PBS at pH 8, 0.8% Triton X-100, 10 mM DTT, 5 mM EDTA, and complete protease inhibitor Roche). The homogenates were centrifuged (13,000 × g, 4°C, 10 min) and the supernatants were removed. The supernatants were assayed using the Bio-Rad protein assay to determine the loading concentration. The supernatants were then mixed with a loading buffer, incubated at 55°C for 15 min, and separated by SDS-PAGE (4–15% Mini-PROTEAN TGX Stain-Free, Bio-Rad,). The Trans-Blot Turbo Transfer System (Bio-Rad) was used to transfer proteins onto Polyvinylidene Fluoride (PVDF) membranes. The total protein concentration for the transfer efficiency and blot normalisation was assessed using the stain-free system. This involved exposing the gels to UV and imaging before and after transfer using the ChemiDoc MP System (Bio-Rad). For JPH, the staining blots were incubated with rabbit anti-JPH antibody [1: 1,000, custom made (Van-Oort et al., 2011)] overnight at 4°C, and then incubated with the goat anti-rabbit Alexa Fluor 647 antibody (1: 20,000, ThermoFisher) for 1 h at room temperature. For RyR, the staining blots were incubated with the mouse anti-RyR antibody (1:500, MA3-916, ThermoFisher) overnight at 4°C, and then incubated with the goat anti-mouse Alexa Fluor 647 antibody (1:20,000, ThermoFisher) for 1 h at room temperature. The blots were imaged at 700 nm with a 1 min integration time using the Odyssey Fc system (LI-COR Biosciences, Nebraska, USA).

### Statistics

A Linear Mixed-Effects (LME) model was used to analyse the hierarchical nature of our data for colocalisation and RyR cluster parameters. For comparisons between NF and IDCM patients, the fixed effects were in the disease status (IDCM, NF) and cell region (t-tubules, sarcolemma, corbular) and the random effects were in the heart. For the analysis of the t-sheets, the fixed effects were in the cell region (t-sheets, t-tubules, none) and the random effects were in the heart. *A post-hoc* comparison of the marginal means was performed with a Sidak multiple comparisons test. For the assessment of the RyR cluster size and JPH counts a least-squares linear regression was used. For the comparison of western blot data, a two-tail *t*-test was utilised. Statistical tests were carried out in IBM SPSS Statistics 25.

## Results

### Nanoscale Distribution of RyR and JPH

**Figure 1:**
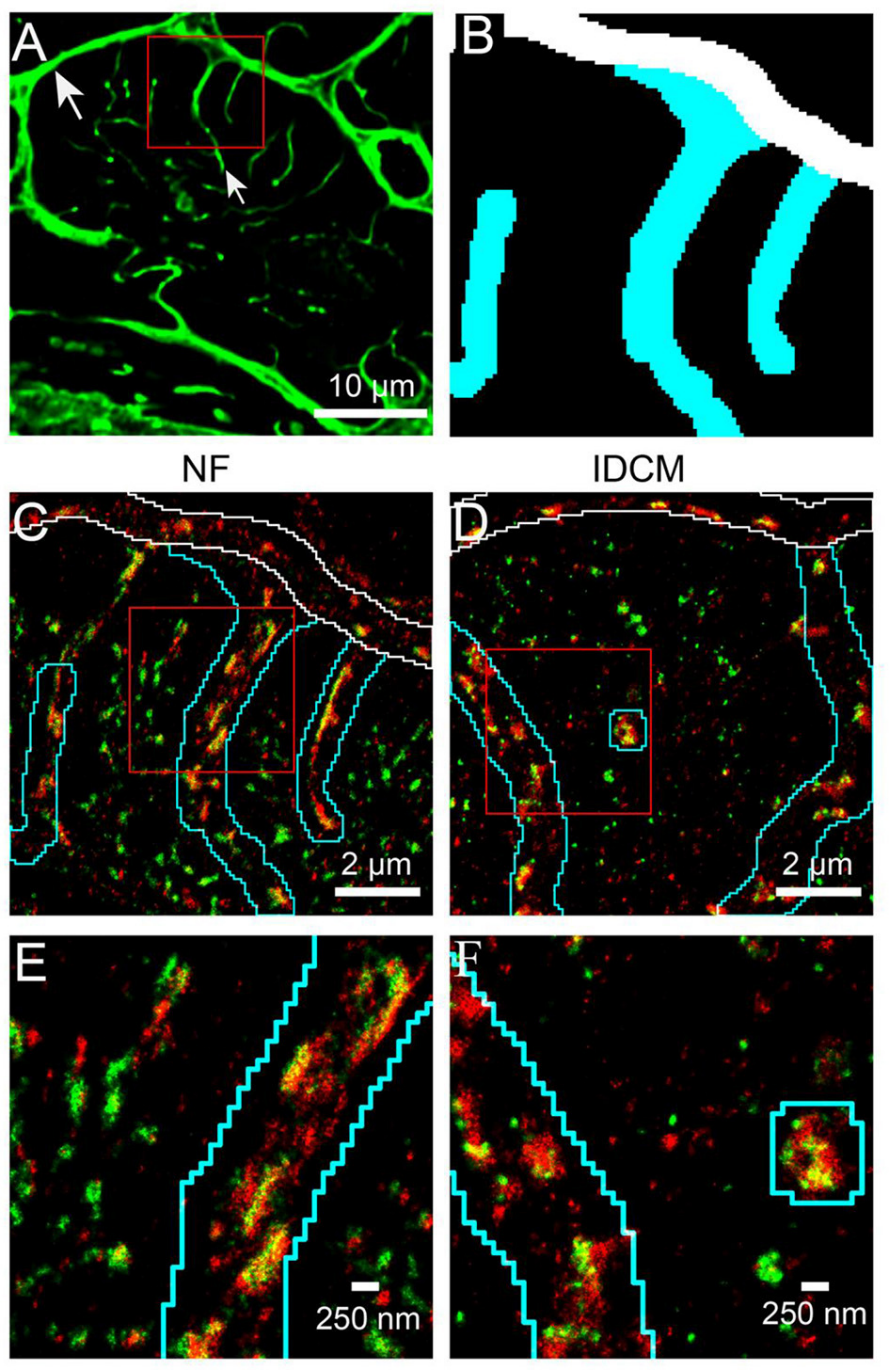
Super-resolution imaging of RyR and JPH in donor and IDCM human cardiomyocytes segmented into t-tubular, sarcolemmal and corbular SR regions. **(A)** Confocal imaging of the WGA labelled donor cardiac myocyte (transversely orientated). The WGA labelling identifies the sarcolemma (large arrow) and t-tubules (small arrow). **(B)** Binary mask of regions near t-tubules (cyan), surface sarcolemma (white) and corbular SR (Black) generated from the WGA labelling of the red box in “**(A)**”. **(C)** Super-resolution imaging of RyR (green) and JPH (red) in donor myocyte indicated by the red box in “**(A)**”. The outline of the t-tubule mask is shown in cyan and the sarcolemma mask in white. Cellular areas outside these outlines contain the corbular SR. **(D)** Super-resolution image of RyR and JPH in an IDCM myocyte, outlines for t-tubule and sarcolemma masks as in “**(C)**”. **(E)** Enlargement of the red box from donor myocyte in “**(C)**”. **(F)** Enlargement of the red box from IDCM myocyte in “**(D)**”.

Super-resolution imaging was used to examine the nanoscale distribution of RyR and JPH in cardiac myocytes from NF and IDCM hearts (Figure 1). The distribution of these proteins was separated into cellular regions based on correlative confocal imaging as previously described (Soeller et al., 2017). This segmentation was achieved by labelling the sections with WGA that labels the t-tubules, sarcolemma, and extra-cellular matrix (Figure 1A). A binary mask was then created from the WGA labelling to separate the cell into the following cellular regions; t-tubule, surface sarcolemma, and corbular (Figure 1B). Visual inspection of the RyR and JPH labelling suggests there is a close association between the two proteins at the t-tubules and surface sarcolemma. The paradoxical appearance of RyR and JPH labelling within the t-tubule mask is due to the ~700 nm Z resolution of 2D dSTORM. Within the corbular regions, there was an evident spatial separation in the RyR and JPH labelling compared with the other regions. Upon the visual inspection of the RyR and JPH distribution, there was no observable difference between the NF and IDCM human hearts.

**Figure 2:**
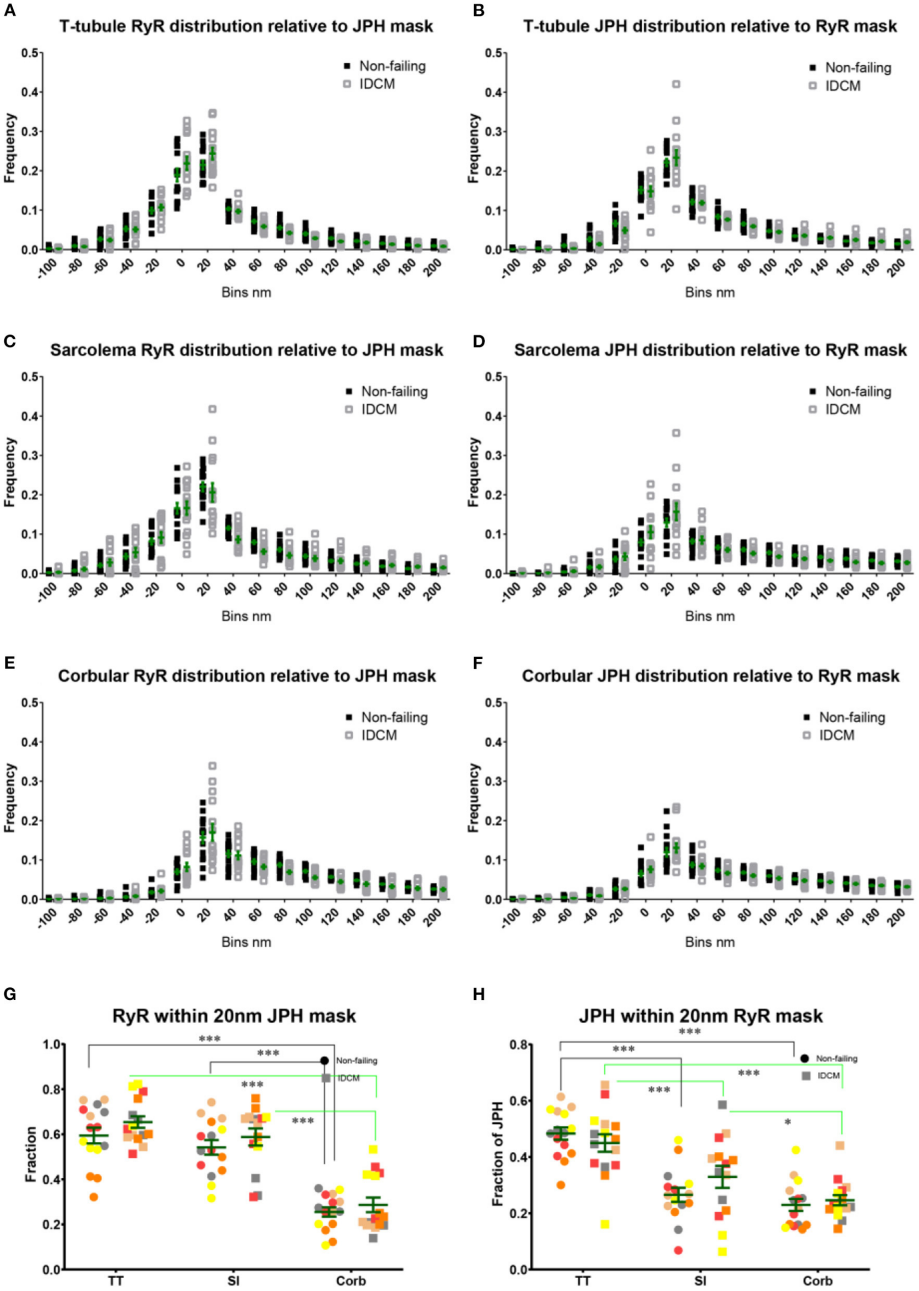
Nanoscale distribution of RyR and JPH labelling in donor and IDCM human cardiac myocytes at t-tubular, sarcolemmal and corbular regions. **(A)** Frequency plot of RyR distribution relative to the mask of JPH labelling at the T-tubule regions. **(B)** Frequency plot of JPH distribution relative to the mask of RyR labelling at the T-tubule regions. **(C)** Frequency plot of RyR distribution relative to the mask of JPH labelling at the sarcolemmal regions. **(D)** Frequency plot of JPH distribution relative to the mask of RyR labelling at the sarcolemmal regions. **(E)** Frequency plot of RyR distribution relative to the mask of JPH labelling at the corbular SR regions. **(F)** Frequency plot of JPH distribution relative to the mask of RyR labelling at the corbular SR regions. **(G)** Total fraction of RyR with 20 nm of JPH mask for the t-tubular, sarcolemma and corbular SR regions. **(H)** Total fraction of JPH with 20 nm of RyR mask for the t-tubular, sarcolemma and corbular SR regions. Distributions were estimated from *n* = 5 donor hearts, *n* = 5 IDCM hearts, 3 cells were analysed from each heart. For “**(G,H)**” groups linked with a line are significantly different at either *p* < 0.05 and *p* < 0.001 as indicated by ^*^ and ^***^ respectively. LME model was used to test for the effects of the disease status (IDCM, NF) and cell region (t-tubules, sarcolemma, corbular) and random effects were in the heart. *P*-values reported are *post-hoc* comparisons of marginal means using a Sidak test for multiple comparisons. Symbols of the same colour in “**(G,H)**” are from the same heart.

A distance-based colocalisation analysis was used to quantify the relative nanoscale distribution of RyR and JPH in each of the three cellular compartments (Figure 2). This involved calculating the amount of RyR labelling as a function of distance, in 20 nm bins, from the edge of JPH labelling. The reverse, i.e., the amount of JPH labelling as a function of distance from the edge of RyR labelling, was also calculated. For simplicity, these parameters are herein referred to as RyR or JPH colocalisation, respectively. This distance-based analysis demonstrated a greater RyR colocalisation at the t-tubules and sarcolemma compared with the corbular regions (Figures 2A,C,E). A similar colocalisation pattern was seen for JPH (Figures 2B,D,F) confirming the visual impression of the labelling patterns. The ability to quantify the distribution of each protein in 20 nm bins allowed for selecting a biological relevant distance range to measure the total colocalisation for each protein. For the RyR and JPH, a total fraction of label within 20 nm of the mask of the other channel was chosen, based on the ~27 nm size of RyRs (Wagenknecht et al., 1989). The total RyR and JPH colocalisation were calculated from the distance-based histogram analysis and are presented in Figures 2G,H, respectively. The Linear Mixed-Effects (LME) modelling found no change in the total RyR colocalisation between the NF and IDCM myocytes (0.46 ± 0.036 and 0.51 ± 0.036, respectively) but found a change in the total RyR colocalisation between the t-tubule, sarcolemma, and corbular regions (0.62 ± 0.029, 0.56 ± 0.029, and 0.27 ± 0.029, respectively, *p* < 0.001). A *post-hoc* pairwise comparison of the marginal means, in general, confirmed the findings from the LME model and are presented in Figure 2G. The total JPH colocalisation pattern was similar with LME model, finding no difference between the NF and IDCM myocytes (0.33 ± 0.028 and 0.34 ± 0.028, respectively) but a difference between the regions (0.47 ± 0.023, 0.3 ± 0.023, 0.24 ± 0.023, *p* < 0.001). A *post-hoc* pairwise comparison of the marginal means, in general, confirmed the findings from the LME model and are presented in Figure 2H. In summary, there was increased total colocalisation of both the RyR and JPH in the t-tubule and sarcolemma regions compared with the corbular regions in both NF and IDCM myocytes.

### RyR Cluster Analysis

**Figure 3:**
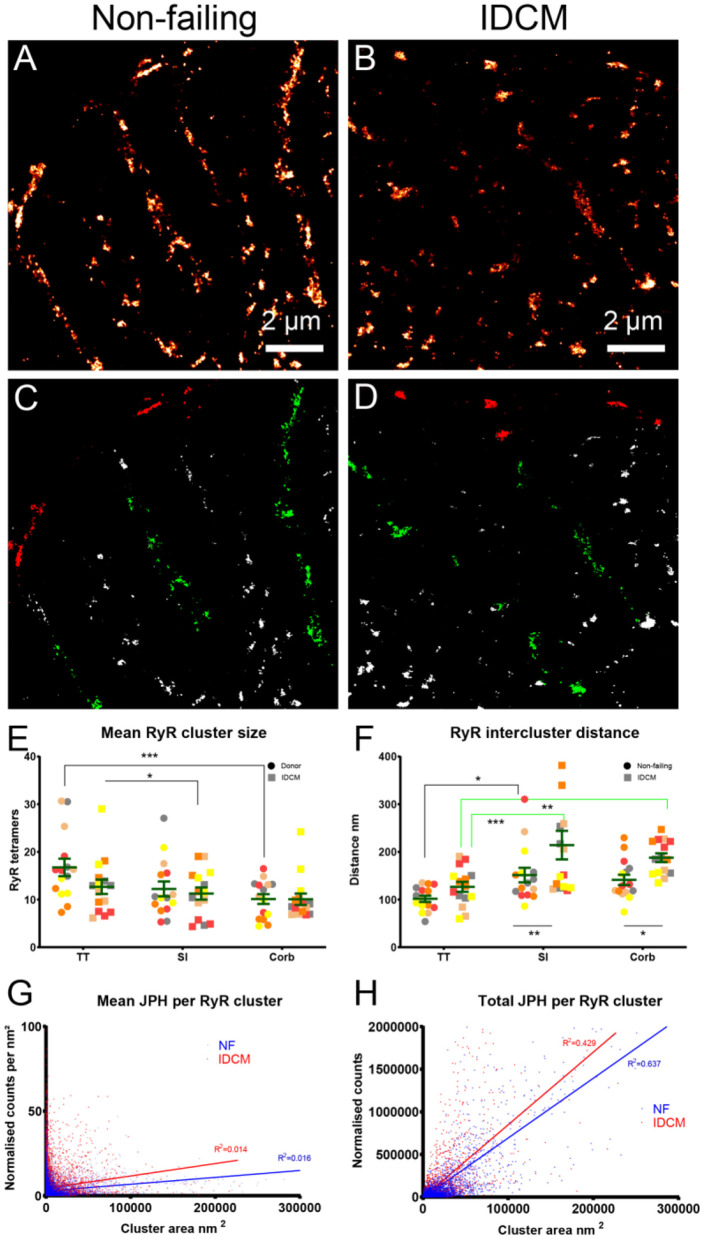
Super-resolution imaging of RyR clusters in donor and IDCM human hearts. **(A)** RyR clusters in Donor myocyte. **(B)** RyR clusters in IDCM myocyte. **(C)** Binary mask of “**(A)**” segmented into TT (green), Sl (red), and Corb regions (white). **(B)** RyR clusters in IDCM myocyte. **(D)** Binary mask of “**(B)**” segmented into TT, Sl, and Corb regions. **(E)** Mean RyR clusters size in NF and IDCM hearts. **(F)** Mean RyR intercluster distance in NF and IDCM hearts. Symbols of the same colour in “**(E,F)**” are from the same heart. **(G)** Mean number of JPH counts per RyR cluster. **(H)** Total number of JPH counts per RyR cluster. Groups linked with a line are significantly different in *post-hoc* pairwise comparison at either *p* < 0.05, *p* < 0.01, and *p* < 0.001 as indicated by ^*^, ^**^, and ^***^ respectively. LME model was used to test for the effects of disease status (IDCM, NF) and cell region (t-tubules, sarcolemma, corbular) and random effects were in the heart. *P*-values reported are *post-hoc* comparisons of marginal means using a Sidak test for multiple comparisons.

The RyR cluster size, RyR supercluster size and nearest neighbour distances between RyR clusters were estimated from the super-resolution images (Figure 3, Supplementary Figure 2A). For this analysis, The RyR images were converted to a binary mask of RyR clusters and were partitioned into the t-tubule, sarcolemma and corbular regions based on the masks generated from the correlative confocal WGA images (Figures 3D,E). The analysis of the RyR cluster sizes demonstrated an exponential distribution at the t-tubule, sarcolemma, and corbular regions with ~60% of clusters containing less than five RyR tetramers with no appreciable difference in the distribution between the NF heart myocytes and IDCM myocytes (Supplementary Figures 1A,C,E). The LME model found no significant change in RyR cluster size between the NF and IDCM myocytes (13 ± 1.7 and 11 ± 1.7, respectively) but statistically significant (*p* < 0.001) differences were identified between the t-tubule, sarcolemma, and corbular regions (15 ± 1.4, 12 ± 1.4, and 10 ± 1.4). The *post-hoc* pairwise comparisons of the marginal means for this analysis are presented in Figure 3E. The LME model revealed no significant change in the RyR supercluster size between the NF and IDCM myocytes (18 ± 2.4 and 15 ± 2.4, respectively) but statistically significant (*p* < 0.001) differences were identified between the t-tubule, sarcolemma, and corbular regions (22 ± 1.9, 6 ± 1.9, and 13 ± 1.9). The *post-hoc* pairwise comparisons of the marginal means for this analysis are presented in Supplementary Figure 2A. The nearest neighbour distances between the RyR clusters also followed an exponential distribution at the t-tubule, sarcolemma, and corbular regions with ~60% of the inter-cluster distances closer than 140 nm (Supplementary Figures 1B,D,F). The LME model analysis of inter RyR cluster distances revealed a significant (*p* = 0.02) difference between the NF and IDCM myocytes (131 ± 12, and 176 ± 12, respectively) and a significant (*p* < 0.001) difference between the t-tubule, sarcolemma, and corbular regions (114 ± 12, 182 ± 12, and 163 ± 12, respectively). The *post-hoc* pairwise comparisons between the groups are presented in Figure 3F. A smaller inter RyR cluster distance was identified at the sarcolemma and corbular SR in non-failing myocytes compared with IDCM myocytes (*p* < 0.01 and *p* < 0.5, respectively). The linear regression between the RyR cluster size and the mean JPH events within each cluster showed a poor fit in both the NF and IDCM myocytes (Figure 3G). However, a good fit was found between RyR cluster size and total JPH events in each cluster (Figure 3H). In summary, the RyR cluster sizes were similar in the NF and IDCM myocytes but reduced intercluster distances were observed in the NF myocytes relative to IDCM myocytes.

### Quantification of RyR Expression

**Figure 4:**
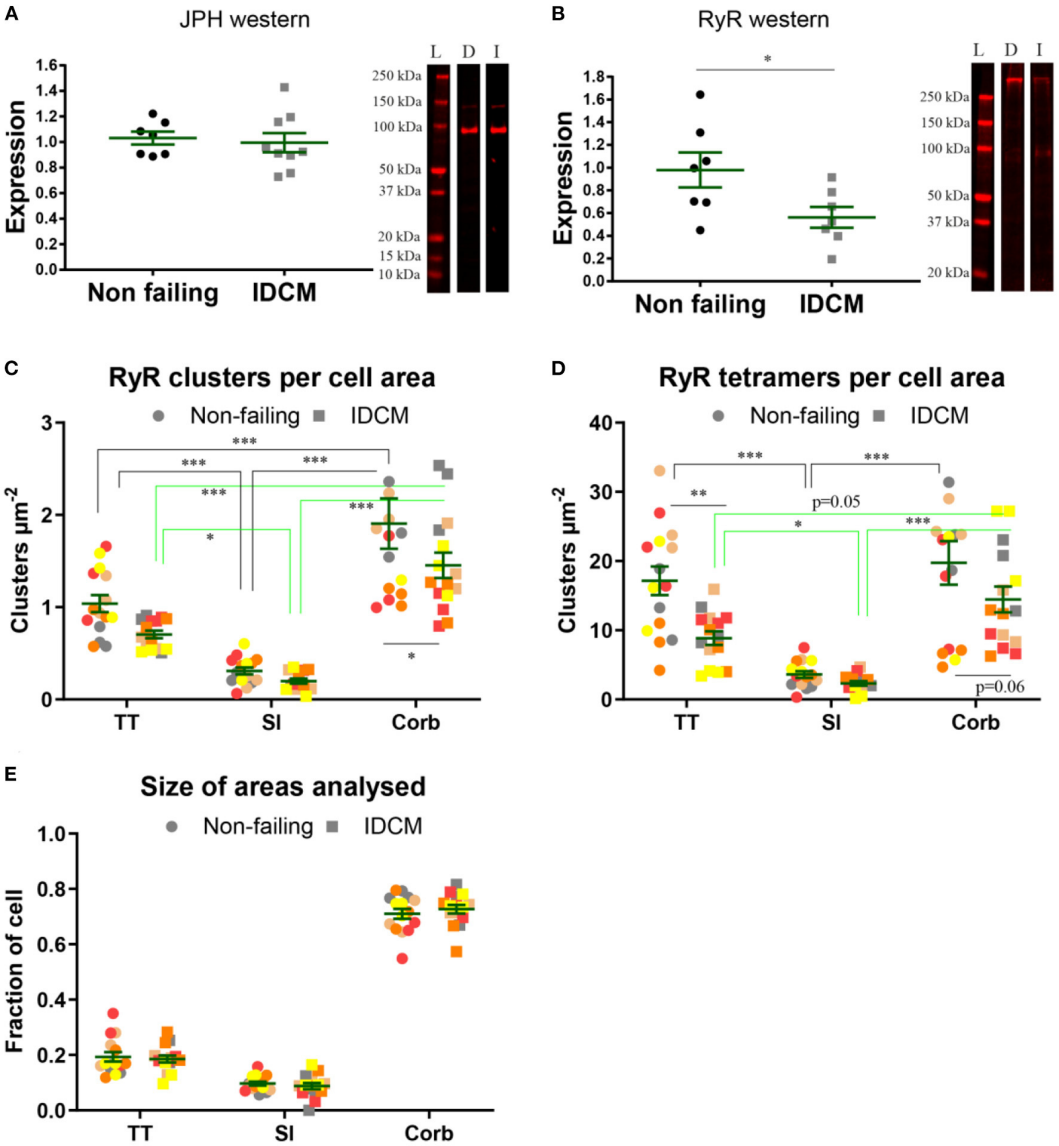
Quantification of expression of JPH and RyR in normal and IDCM hearts. **(A)** JPH western, two-tailed *t*-test *p* = 0.79 *n* = 8 donor, and *n* = 9 IDCM hearts. **(B)** RyR western, two-tailed *t*-test *p* = 0.02^*^ *n* = 8 donor, and *n* = 7 IDCM hearts. For each western exemplar, lanes are shown. L, ladder; D, donor; and I, IDCM. **(C)** Mean number of RyR clusters, *n* = 5 donor, *n* = 5 IDCM hearts, 3 cells were analysed from each heart. The number of clusters per cell area was calculated for TT, Sl, Corb regions. **(C)** Mean number of RyR clusters per cell area coming from TT, Sl, and Corb regions, *n* = 5 non-failing, *n* = 5 IDCM hearts, 3 cells were analysed from each heart. *P* < 0.05^*^ for Corb. **(D)** Mean number of RyR tetramers per cell area coming from TT, Sl, and Corb regions, from *n* = 5 non-failing, *n* = 5 IDCM hearts, 3 cells were analysed from each heart. *P* < 0.05^*^ for TT and *P* = 0.7 for Corb. For “**(C,D)**” Groups linked with a line are significantly different at either *p* < 0.05, *p* < 0.01, and *p* < 0.001 as indicated by ^*^, ^**^, and ^***^ respectively. LME model was used to test for the effects of the disease status (IDCM, non-failing) and cell region (t-tubules, sarcolemma, corbular) and random effects were in the heart. *P*-values reported are *post-hoc* comparisons of marginal means using a Sidak test for multiple comparisons. **(E)** Size of the area fractions TT, Sl, and Corb analysed in “**(C,D)**”. Note normalisation per cell area in “**(C,D)**” means the number of clusters (or tetramers) per cell region, e.g., t-tubules was normalised to total cell area, as an accurate estimation of t-tubular area for normalisation was not possible from diffraction-limited confocal imaging. Symbols of the same colour in “**(C–E)**” are from the same heart.

The western blot analysis revealed no change in the expression of JPH in the NF and IDCM left ventricle but there was a ~2-fold decrease in the RyR expression in the IDCM tissue (*p* = 0.02, Figures 4A,B). To explore where these differences occurred within the cardiac myocytes, super-resolution images were quantified for the number of RyR clusters per cell area and the number of RyR tetramers per cell area (Figures 4C,D). The normalisation per cell area means that the number of clusters (or tetramers) per cell region, e.g., t-tubules, was normalised to total cell area, as an accurate estimation of the t-tubular area for normalisation was not possible from the diffraction-limited confocal imaging. The LME model showed that the mean RyR cluster density between the NF and IDCM myocytes was similar (1.1 ± 0.15 and 0.77 ± 0.15 clusters μm^−2^, respectively) whereas, there were differences between the t-tubule, sarcolemma and corbular regions (0.9 ± 0.13, 0.26 ± 0.13, 1.7 ± 0.13, clusters μm^−2^, respectively, *p* < 0.001). The *post-hoc* pairwise comparisons between the groups are presented in Figure 4C. The mean RyR tetramer density, however, was higher in the NF myocytes compared with IDCM myocytes in the LME model (14 ± 1.7 and 8.4 ± 1.7 tetramers μm^−2^, respectively, *p* = 0.03). Furthermore, there were changes between the t-tubule, sarcolemma and corbular regions (13 ± 1.5, 2.9 ± 0.16, 17 ± 1.5, clusters μm^−2^, respectively, *p* < 0.001). The *post-hoc* pairwise comparisons between the groups are presented in Figure 4D, notably, a decrease in RyR tetramers was found in the IDCM myocytes compared with NF myocytes at the t-tubular regions (*p* < 0.01). From these data, the main effect observed from analysing the super-resolution images was a reduction in the RyR tetramer density at the t-tubules in IDCM cells that correlates with the observed reduction of RyR expression in western blots in Figure 4.

### 3D Confocal Imaging of T-Sheets in IDCM Cardiac Myocytes

**Figure 5:**
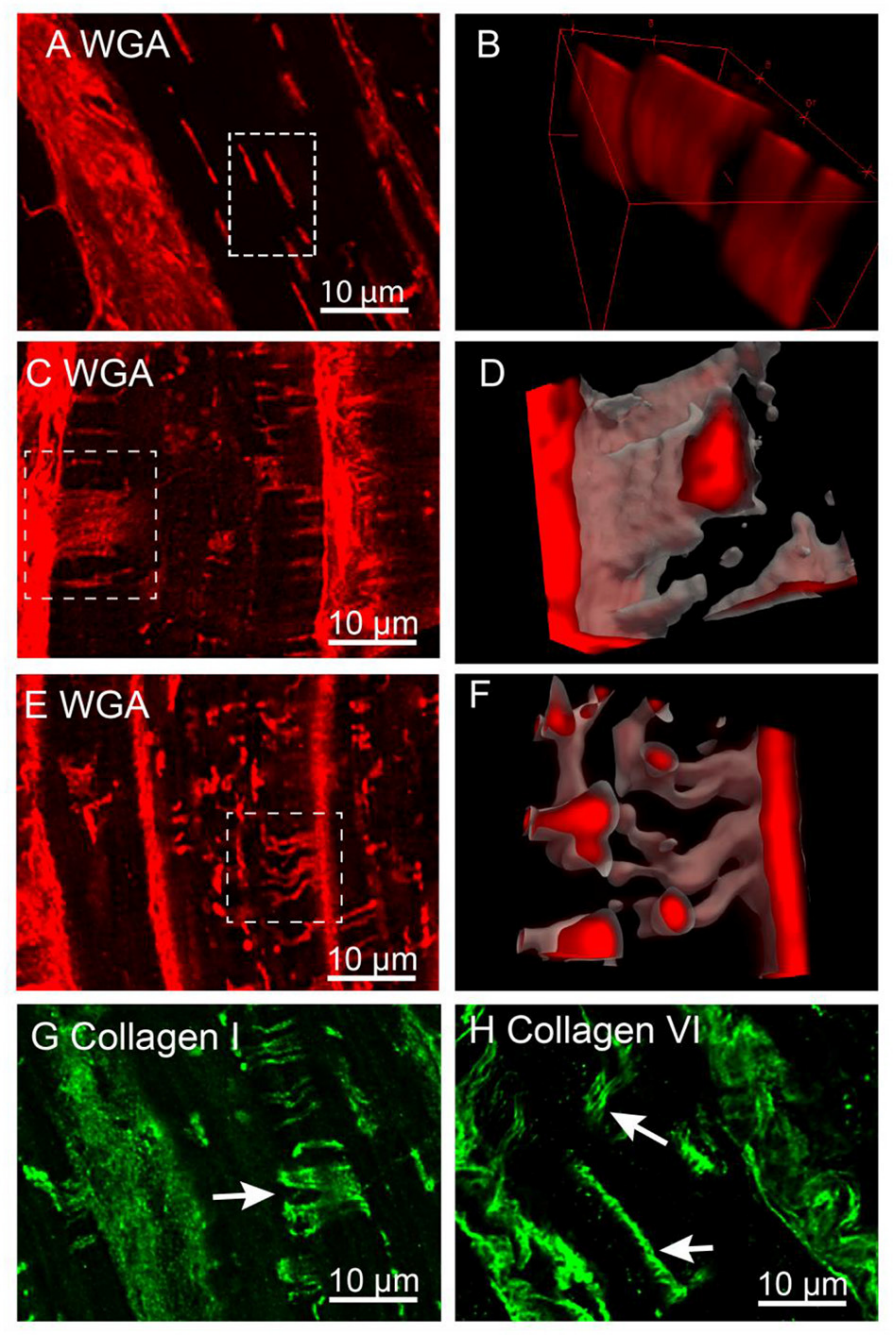
Confocal imaging of t-sheets in the IDCM human heart. **(A)** WGA labelling of t-sheets that superficially appear as axial tubules in the single-plane image. **(B)** 3D projection of the white rectangular region from “**(A)**” confirm the presence of sheet-like structures. **(C)** Confocal stack Z-projection 1.5 μm deep of in-plane WGA labelled t-sheet (white arrow). **(D)** 3D projection of t-sheet from “**(C)**”. **(E)** Confocal stack Z-projection 1.5 μm deep of WGA labelling indicating the presence of two t-tubules that have appeared to fuse (white arrow). **(F)** 3D projection of fused t-tubules from “**(E)**”. **(G)** Confocal stack Z-projection 1.5 μm deep of collagen I labelling of t-sheets (white arrow). **(H)** Confocal stack Z-projection 1.5 μm deep of collagen VI labelling of t-sheets (white arrows). For each label *n* = 5 IDCM hearts, 3 cells were imaged from each heart.

To investigate the structure of t-sheets, we used confocal microscopy due to its ability to capture 3D data in an extended volume, whereas, typically 3D dSTORM imaging is limited to a small axial range of ~1 μm deep. This is due to the out of focus bleaching limiting the capture at multiple Z-positions. The confocal Z-stacks, ~10 μm deep, of WGA labelled tissue were analysed to determine if the recently described t-sheets were present in the IDCM human heart samples (Figure 5). We did not observe t-sheets within the NF hearts. In all five IDCM hearts examined, the membrane structures exhibiting the characteristics of t-sheets were readily observed, i.e., large flattened membrane invaginations that display strong WGA labelling and have a sheet-like appearance when examined in 3D images. Figure 5A is an example of a cell with apparent “axial” t-tubules in a 2D view. However, close inspection of the confocal Z-stack demonstrated that these “axial” tubules persist for several microns within the image volume. A 3D rendering (Figure 5B) of the highlighted region reveals that these are sheet-like structures that are comparable to the t-sheets described by Seidel et al. (2017a). Furthermore, the careful examination of WGA-labelled IDCM heart tissue identified examples of t-sheets that extended along the lateral imaging plane of the confocal microscope (white arrows Figures 5C,D). At the higher resolution provided by in-plane imaging, it was observed that t-sheets are composed of multiple apparently “fused” t-tubules. Also, there are numerous regions where two or more adjacent t-tubules appeared to intertwine and become fused (white box Figures 5E,F).

Previously, we demonstrated that dilated t-tubules in IDCM hearts contain increased amounts of collagen (Crossman et al., 2017). To determine that collagen was a component of t-sheets, tissue sections were labelled for collagen types I and VI. The confocal Z-stacks of these tissue samples demonstrated sheet-like structures made up of multiple fibres of both types I and VI collagen (white arrows Figures 5G,H) consistent with the conclusion that the t-sheet luminal spaces also contain collagen.

**Figure 6:**
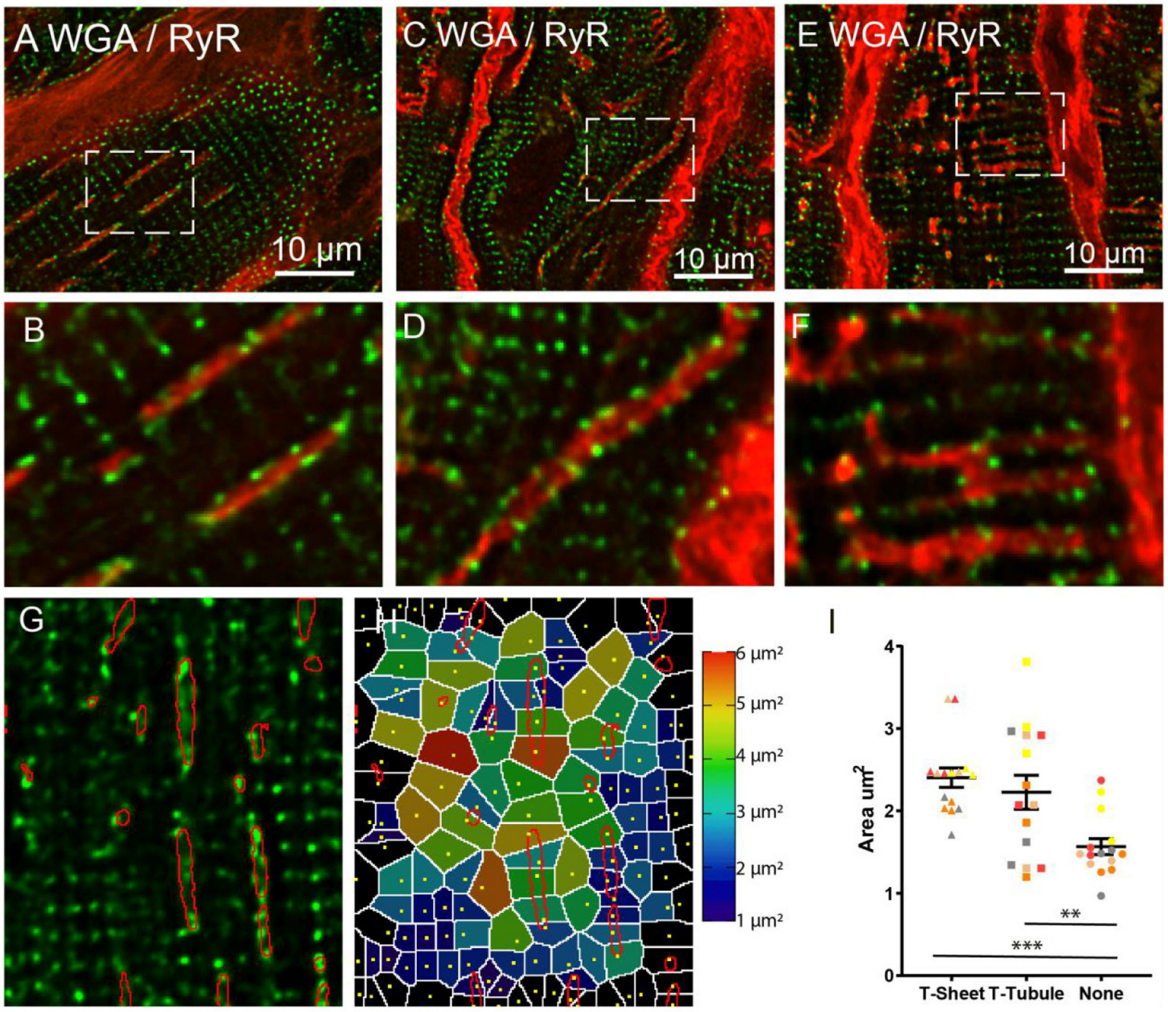
Confocal imaging of WGA labelled t-sheets (red) and ryanodine receptors (green) in the IDCM human heart. **(A)** 1.5 μm Z-projection of t-sheets perpendicularly orientated to the image plane. The white arrow points to the plane image of the sarcolemma showing WGA labelling and RyR clusters. **(B)** Enlargement of the region in “**(A)**”, note area between adjacent t-sheets lacking RyR. **(C)** T-sheet/enlarged oblique running t-tubule. **(D)** Enlargement of the region in “**(B)**”, note area between adjacent to t-sheet/t-tubule lacking RyR. **(E)** Dilated t-tubules. **(F)** Enlargement of the white box in “**(E)**” showing fused t-tubules. **(G)** Single plane confocal image of region RyR labelling with t-sheet masks highlighted in red. **(H)** Voronoi diagram showing the area covered by each RyR cluster, RyR cluster location indicated by yellow dots. **(I)** The average Voronoi areas from regions with t-sheets, t-tubules, and no WGA labelling (None). For each region *n* = 5 IDCM hearts, 3 cells were imaged from each heart. ^**^p < 0.01 and ^***^p < 0.001. LME model was used to test for effects of the region (t-sheets, t-tubules, and no WGA labelling) and random effects were in the heart. *P*-values reported are *post-hoc* comparisons of marginal means using a Sidak test for multiple comparisons. Symbols of the same colour in “**(I)**” are from the same heart.

To assess the organisation of RyR Ca^2+^ release clusters in relation to the t-sheets (Figure 6) confocal Z-stacks of WGA and RyR labelled HF tissue were captured. The RyR clusters were apparent and immediately adjacent to the t-sheets and appeared to follow the outline of the WGA labelling that delineates the t-sheets (Figure 6B). This was particularly apparent in the large oblique t-tubules where the normal striated appearance of RyR cluster rows was disrupted with the RyR clusters following the diagonal direction of the WGA labelling (Figures 6C,D). In the other enlarged but less prominently remodelled t-tubules, a similar association between the RyR clusters and WGA labelling was observed (Figures 6E,F). Of particular note, there were areas devoid of RyR clusters near the t-sheets and t-tubules (Figure 6G) that disrupted the usual sarcomeric grid-like pattern. To quantify these visual observations, we analysed the RyR clusters in regions associated with t-sheets, t-tubules and with no WGA. This involved determining RyR cluster centroids; from these centroids, a Voronoi diagram was generated which determined the area of pixels that were closest to the particular centroid relative to other centroids (Figure 6H). The associated Voronoi areas provided an index that is inversely proportional to local RyR density. The LME model revealed a difference in the mean Voronoi areas between the t-sheet, t-tubule and regions without WGA staining (2.4 ± 0.1, 2.2 ± 0.1, and 1.6 ± 0.1, respectively, *p* < 0.001). A *post-hoc* comparison of the marginal means demonstrated that the t-sheets and t-tubule regions had greater Voronoi areas (i.e., lower local RyR density) than regions without WGA labelling (*p* < 0.001 and 0.01, respectively, Figure 6I).

### Super-Resolution Imaging of RyR Clusters at T-Sheets

**Figure 7:**
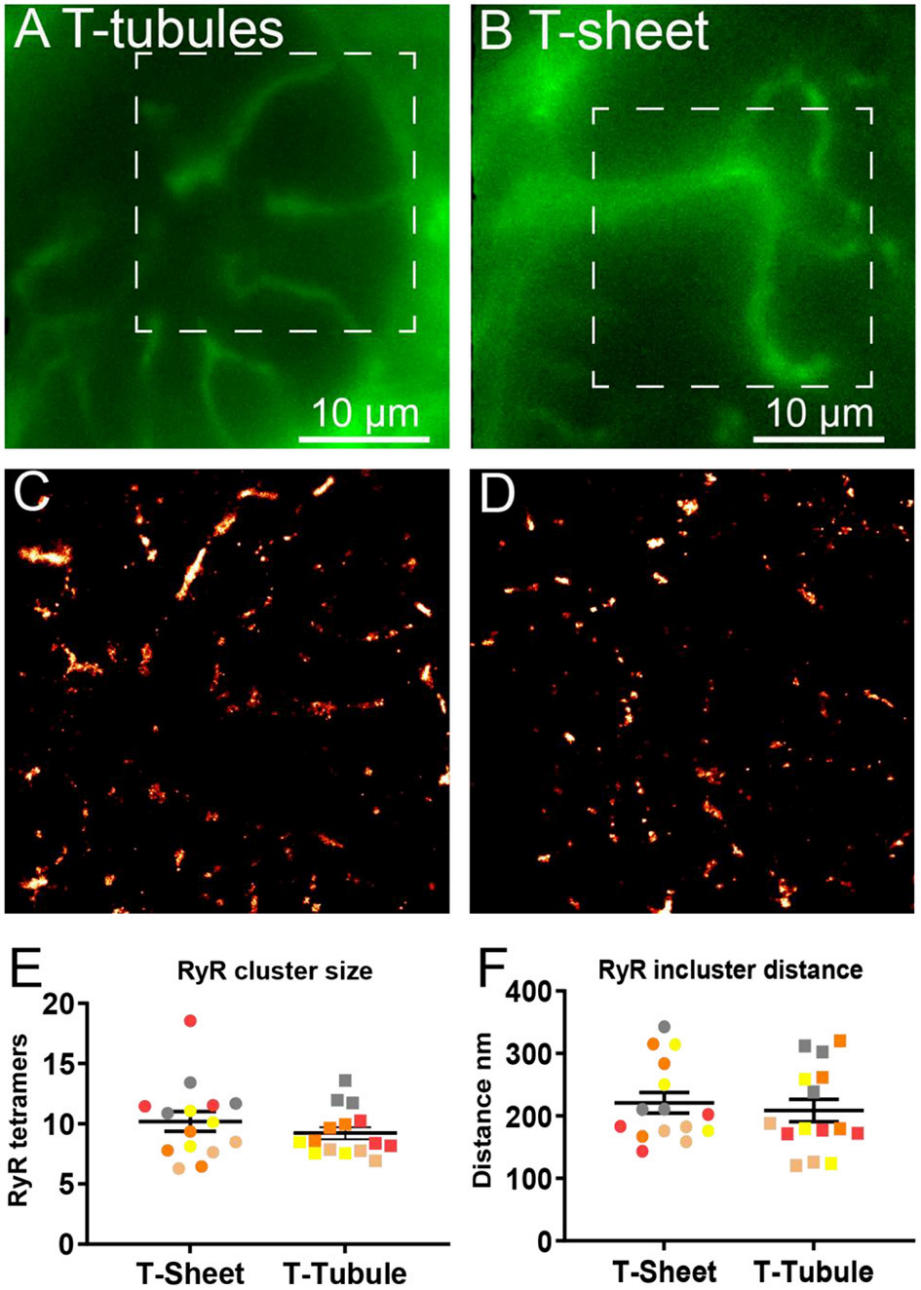
Comparison of RyR clusters between t-tubules and t-sheets regions in IDCM human hearts. **(A)** Epifluorescence image of WGA labelled t-tubules recorded on super-resolution microscope before subsequent nanoscale imaging of RyR clusters. **(B)** Epifluorescence image of WGA labelled t-sheet recorded on super-resolution microscope before subsequent nanoscale imaging of RyR clusters. The t-sheet identification was confirmed by changing the focus by several microns and the structure designated a t-sheet if it was visible throughout the change in focus. **(C)** Super-resolution image of RyR clusters from t-tubular region recorded from the white box in “**(A)**”. **(D)** Super-resolution image of RyR clusters from t-sheet region recorded from the white box in “**(B)**”. **(E)** Mean RyR cluster size in the t-tubule and t-sheet regions. **(F)** Mean RyR cluster nearest neighbour distance in the t-tubule and t-sheet regions. Five IDCM hearts were analysed with 3 t-tubule and 3 t-sheet regions imaged from each heart. LME model was used to test for effects of the region (t-sheets and t-tubules) and random effects were in the heart. For RyR cluster size *p* = 0.53 and for RyR cluster nearest neighbour *p* = 0.19. Symbols of the same colour in “**(E,F)**” are from the same heart.

We used super-resolution microscopy to provide a nanoscale analysis of the RyR clusters at both the t-sheet and t-tubule regions of the IDCM myocytes to determine if t-sheet remodelling drives nanoscale changes in the RyR cluster organisation (Figure 7). Epifluorescence microscopy of WGA labelling was first used to identify transversely orientated cell regions containing either t-tubules or t-sheets (Figures 7A,B). In the same region, super-resolution microscopy was then used to acquire images of the RyR clusters (Figures 7C,D). The analysis of the RyR cluster sizes revealed an exponential distribution with ~60% of the clusters containing less than five RyR tetramers with no appreciable difference in the distribution between the t-tubule and t-sheet regions (Supplementary Figure 1G). The LME model of the mean RyR cluster sizes demonstrated no statistical difference between t-tubules and t-sheets (Figure 7E). Similarly, superclusters were not statistically different between t-tubules and t-sheets (Supplementary Figure 2B). The nearest neighbour distances between the RyR clusters also followed an exponential distribution with ~60% of the inter-cluster distances closer than 140 nm (Supplementary Figure 1H). The LME model of the mean RyR nearest neighbour distances demonstrated no statistical difference between t-tubules and t-sheets (Figure 7F).

## Discussion

### RyR and JPH Co-Localisation

We found that there is an increased co-localisation between RyR and JPH at the t-tubules and sarcolemmas compared with corbular regions. This finding is consistent with the role of JPH in forming cardiac junctions by providing a structural link between the SR and sarcolemma (Takeshima et al., 2000). However, contrary to our *a priori* hypothesis, we found no change in the co-localisation of the RyR with the JPH between the donor and end-stage IDCM myocytes. Furthermore, the western blot analysis showed no change in JPH expression. These data suggest that changes in JPH expression are not primarily responsible for t-tubule remodelling in the IDCM hearts we examined. Previously, the knockdown of JPH expression in mice reduced the colocalisation of RyR and JPH (Munro et al., 2016), promoted t-tubule remodelling and resulted in the loss of cardiac junctions, and impaired calcium release that resulted in acute HF (Van-Oort et al., 2011). Furthermore, downregulation of JPH has been observed in several animal models and in human HF including dilated cardiomyopathy (DCM) (Minamisawa et al., 2004; Landstrom et al., 2007; Wei et al., 2010; Zhang et al., 2013; Guo et al., 2015; Wang et al., 2018; Xiao et al., 2018).

These conflicting observations, explained by the downregulation of JPH in experimental HF, is not a consistent finding and may be model specific. For example, tachycardia-induced HF in sheep and thoracic aortic banding in ferrets result in HF with t-tubule loss but no change in JPH expression (Caldwell et al., 2014). With regards to human HF, the samples in our study are clinically described as idiopathic DCM whereas in other publications were described as non-specified DCM (Zhang et al., 2013; Guo et al., 2015; Xiao et al., 2018). It is thus, possible, that the DCM samples between the studies represent different aetiologies. DCM is a multifactorial disease that can result from a diverse range of causes including inherited mutations, infection and inflammation, autoimmunity, metabolic syndrome, and peripartum cardiomyopathy (Schultheiss et al., 2019). Idiopathic and inherited forms are the most commonly recorded cases of DCM. However, to accurately identify a case as idiopathic, it would require the exclusion of all known causes of DCM, which presents a diagnostic challenge, particularly as the availability of the required diagnostic procedures vary widely between hospitals (Schultheiss et al., 2019). The data shown here indicate that in at least some forms of DCM, JPH down-regulation does not appear to be the driving factor in t-tubule remodelling. This conclusion is supported by a recent study that found no change in the mRNA levels of JPH in DCM patients (Frisk et al., 2021). Furthermore, the JPH cluster density did not change between the non-failing and HF hearts, although there was a decrease with age in the non-failing heart (Lyu et al., 2021). JPH has been proposed as a therapeutic target for HF to improve the t-tubule structure and cardiac function (Reynolds et al., 2016). Our data suggest that this may only be effective in certain subpopulations of patients. This highlights the need for personalised medicine where underlying molecular defects can be characterised so that targeted therapies can be utilised effectively. This goal could be achieved by greater utilisation of endomyocardial biopsy and a combination of molecular methods including confocal and super-resolution microscopy to identify the underlying pathology involved (Crossman et al., 2015b).

### RyR Expression and Cluster Geometry

The data presented here indicate an overall reduction in the density of RyR within IDCM cardiac myocytes and may contribute to the reduced Ca^2+^ release as previously documented in myocytes from the failing human heart (Beuckelmann et al., 1992) and should be considered alongside the well-documented defects in sarcoplasmic reticulum Ca^2+^ ATPase SERCA expression in HF (Lipskaia et al., 2010). This conclusion is supported by the ~50% reduction of RyR expression in IDCM hearts as measured by the western blot. Previous analyses have produced conflicting results (Hasenfuss et al., 1997). Our results were confirmed through super-resolution microscopy and the difference between studies may reflect the underlying pathology. Super-resolution imaging indicated that the reduced expression of RyR was predominately due to the decreased RyR cluster density, as previously documented (Crossman et al., 2011) and to a lesser extent, a change in the RyR cluster size. In IDCM, RyR cluster density was lower at the corbular regions as measured by both the increased RyR inter-cluster distance and the decreased number of RyR clusters. An increased distance between non-junction RyR clusters could reduce the ability of the calcium transient to propagate to adjacent non-junctional RyR clusters. This could have a substantial impact as a large portion of RyR clusters are non-junctional in human ventricular myocytes (Crossman et al., 2011; Seidel et al., 2017a) (Figure 4). This contrasts with the rat ventricular myocytes where the junctional clusters dominate (Jayasinghe et al., 2009, 2012b; Scriven et al., 2010) but is closer to the rabbit, pig, and dog ventricular myocytes (Sachse et al., 2009, 2012; Biesmans et al., 2011).

In IDCM myocytes there was a trend for smaller t-tubular RyR clusters (~24%) and reduced t-tubule RyR cluster density (~35%), although this did not reach statistical significance. However, the total t-tubular RyR tetramers per cell decreased by ~50% in the IDCM myocytes, a parameter that includes the effects of both RyR cluster size and density. Decreased t-tubular RyR tetramers would likely reduce the release flux of junctional Ca^2+^ release thereby reducing the amplitude of the Ca^2+^ transient. Overall, the RyR cluster and supercluster size did not differ between the donor and IDCM myocytes. However, there were larger RyR clusters and superclusters at the t-tubular and sarcolemmal regions compared with the corbular regions in NF myocytes. This size difference between the junctional and non-junctional RyR clusters was lost in the IDCM myocytes. Larger junctional RyR clusters size may increase their sensitivity to trigger Ca^2+^ because the larger RyR clusters size was found to increase the frequency of spontaneous sparks in the rat ventricular myocytes (Galice et al., 2018). In summary, the loss of RyR tetramers and reduced cluster sizes at the t-tubules in IDCM could reduce the potential of junctional RyRs to activate. This would be further exacerbated by the increased distances between the non-junctional RyR clusters, decreasing the ability of the cell to generate a robust calcium transient.

### T-Sheets in Human Heart Failure

In agreement with Seidel et al. (2017a), we identified t-sheets in all of our IDCM human hearts. The 3D confocal imaging of the WGA-labelled sections several microns deep allowed the extent of t-sheet structures to be visualised (Figure 5). In general, only parts of the t-sheets could be visualised in single confocal micrographs (Figure 5A) notably when viewed in isolation, i.e., 2D optical sections could be mistaken for axial tubules (Seidel et al., 2017a). Careful examination of the tissue sections captured t-sheets orientated parallel to the imaging plane providing the benefit of a ~3-fold higher lateral resolution (Figure 5C). These images indicated that t-sheets had the appearance of several t-tubules that had fused, compatible with the idea that t-sheets may represent an advanced state of the t-tubule remodelling. Furthermore, there were numerous examples of small or perhaps “immature” t-sheets that contained only two t-tubules that were fused over only part of their lengths (Figure 5D). Consistent with this proposition, more t-sheets were found in patients with prolonged HF, and in patients that failed to functionally recover while mechanically unloaded with a left ventricle assist device (Seidel et al., 2017a). These data suggest that t-sheets are associated with the pathology of advanced HF.

We have previously proposed that increased fibrosis could directly drive the remodelling of t-tubules based on the finding of increased collagen, particularly type VI, within the lumen of the t-tubules in IDCM patients (Crossman et al., 2017). Here, we report multiple reticular fibres (Ushiki, 2002), of both type I and type VI collagen, within the t-sheets indicating that fibrosis could drive both t-tubule and t-sheet remodelling in HF. This suggests a direct link between load, fibrosis, and t-tubule disarray. Consistent with this hypothesis, t-tubule remodelling, including the formation of t-sheets, was associated with local fibrosis in a rabbit model of myocardial infarction (Seidel et al., 2017b). Notably, in that study, the fibrosis was estimated based on WGA labelling. We have previously demonstrated that collagen VI comprises a large component of the WGA signal (Crossman et al., 2017) indicating that this collagen may also be a feature of t-tubule/t-sheet remodelling in this animal model.

### RyR Clusters Organisation at T-Sheets and T-Tubules

The confocal analysis demonstrated that the WGA labelled t-sheets and t-tubules colocalised with numerous RyR clusters. Presumably, these RyR clusters would be directly stimulated by the action potential which travels down the t-tubules. Given the tortuous and highly variable t-sheet and t-tubule architecture in the myocytes of the IDCM myocardium, this would be expected to lead to highly variable and asynchronous Ca^2+^ release and contraction compared to the much more regular organisation of the t-tubules and RyR in the non-failing myocardium (Crossman et al., 2011). Notably, the RyR clusters appeared to follow the morphology of remodelled t-sheets/t-tubules breaking the symmetry of RyR cluster organisation. For example, this was particularly noticeable in regions with oblique running t-tubules (Figure 6B) where there was a diagonal arrangement of RyR clusters along with the WGA labelling that disrupted the usual sarcomere grid-like arrangement of the RyR clusters. These observations indicate that the process that drives the t-tubule remodelling also leads to a disruption in the organisation of the sarcoplasmic reticulum and agrees with previous electron tomography that revealed remodelling of both t-tubules and the sarcoplasmic reticulum in the failing sheep heart (Pinali et al., 2013). Moreover, we noticed that many regions adjacent to the t-sheet and t-tubules in the failing heart had an absence of RyR clusters that was quantified by the enlarged Voronoi areas in these regions. A similar finding has been found for dyssynchronous HF in dogs where increased RyR density has been reported in regions of reduced t-tubule density at cell ends (Li et al., 2015). The Voronoi areas give an estimate of the area that would be dominated by Ca^2+^ released from its respective RyR centroid assuming uniform Ca^2+^ release. Larger Voronoi areas indicate a sparser local density of RyR clusters and these would be expected to contribute to the slowed Ca^2+^ release in these regions, consistent with the previously documented delayed Ca^2+^ release documented in the failing human heart (Beuckelmann et al., 1992). Super-resolution imaging of the RyR cluster geometry found no difference in the RyR cluster size and RyR inter-cluster distances between the cell regions with t-tubules and cell regions with t-sheets (Figure 7) and agrees with the confocal data on the Voronoi cell size, an independent measure of RyR cluster density. Therefore, our data indicate that t-sheet remodelling has a negligible effect on RyR clusters size.

## Limitations

Cardiac junctions have a reported gap of ~10 nm (Takeshima et al., 2000) estimated from electron microscopy measurements, which is less than the ~30 nm resolution we previously reported for our dSTORM microscopy (Baddeley et al., 2011). Therefore, it is not possible to draw a definitive conclusion on the remodelling of cardiac junctions with the methods we employed. Furthermore, our study was based on a 2D analysis that will not resolve the complex 3D geometry previously reported for the RyR imaged in the isolated rat cardiac myocytes (Shen et al., 2019). This is largely a technical issue associated with imaging optically thick tissue sections that will likely be resolved in the future. Moreover, the regions corresponding to the t-tubules, sarcolemma and corbular were estimated from diffraction-limited confocal imaging and therefore reflect a generalised region rather than a precise measurement of these sub-resolution structures.

## Conclusions

Given the previous reports on the expression of JPH in HF, we expected a reduced co-localisation of RyR and JPH in IDCM. However, we found that there was no change in the distribution of RyR and JPH at the nano-scale. In addition, western blot analysis found no change in the JPH protein expression but did report a downregulation of RyR. Analysis of the RyR cluster sizes identified a similar near-exponential size distribution as previously identified in rodents. The average RyR cluster size was not changed in HF but the density of total RyRs was decreased in agreement with western blot data. Confocal microscopy identified that t-sheets were present in all HF examples examined and that these were associated with the presence of reticular collagen fibres within their lumen. Super-resolution imaging demonstrated no difference in RyR clusters in cell regions containing t-sheets compared to cell regions containing t-tubules.

## Data Availability Statement

The raw data supporting the conclusions of this article will be made available by the authors, without undue reservation.

## Ethics Statement

The studies involving human participants were reviewed and approved by the human tissue used in this study was collected with the informed and written consent of transplant patients or from the families of organ donors of non-failing hearts in accordance with the principles in the Declaration of Helsinki. Ethical approval was provided by Health and Disability Ethics Committees New Zealand (NTY/05/08/050/AM05), Human Research Ethics Committees at the University of Sydney (2016/7326), and St Vincent's Hospital (H03/118). The patients/participants provided their written informed consent to participate in this study.

## Author Contributions

DC, CS, and YH contributed to the conception, design, and drafting of the manuscript. YH, DC, JB, XS, OL, AL, SL, CR, and PR contributed to the data acquisition and experiments. DC, YH, CS, and DB contributed to the data analysis. All authors revised and approved the manuscript.

## Conflict of Interest

The authors declare that the research was conducted in the absence of any commercial or financial relationships that could be construed as a potential conflict of interest.

## Publisher's Note

All claims expressed in this article are solely those of the authors and do not necessarily represent those of their affiliated organizations, or those of the publisher, the editors and the reviewers. Any product that may be evaluated in this article, or claim that may be made by its manufacturer, is not guaranteed or endorsed by the publisher.
