# Supplementary material for: Nanoscale Organisation of Ryanodine Receptors and Junctophilin-2 in the Failing Human Heart
Source: Front Physiol. 2021 Oct 8;12:724372. doi: 10.3389/fphys.2021.724372 (PMC8531480; doi:10.3389/fphys.2021.724372)
Supplement: Supplementary file 1 [file Data_Sheet_1.docx]

Supplementary figures


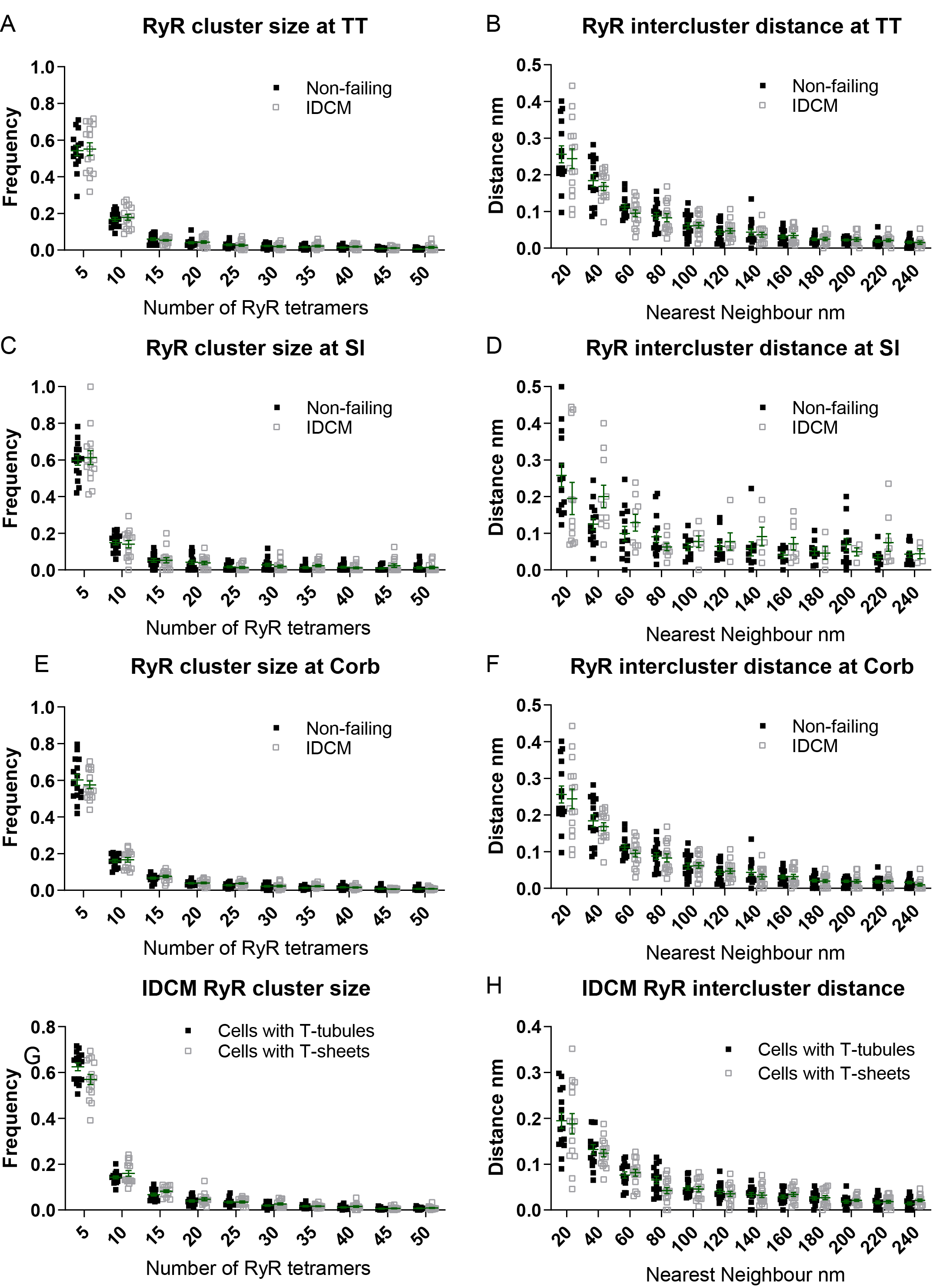


Sup Fig 1. Frequency plots of RyR cluster size and RyR intercluster nearest neighbour distances for donor and IDCM myocytes. A, C, E) Frequency plots for RyR cluster size for t-tubule (TT), sarcolemmal (Sl), and coarbular (Corb) regions respectively. B, D, F) Frequency plot of RyR intercluster nearest neighbour distances for t-tubule (TT), sarcolemmal (Sl), and coarbular (Corb) regions respectively. G) RyR clusters size in IDCM cells with t-tubules or with t-sheets. G) RyR interclusters distances in IDCM cells with t-tubules or with t-sheets.


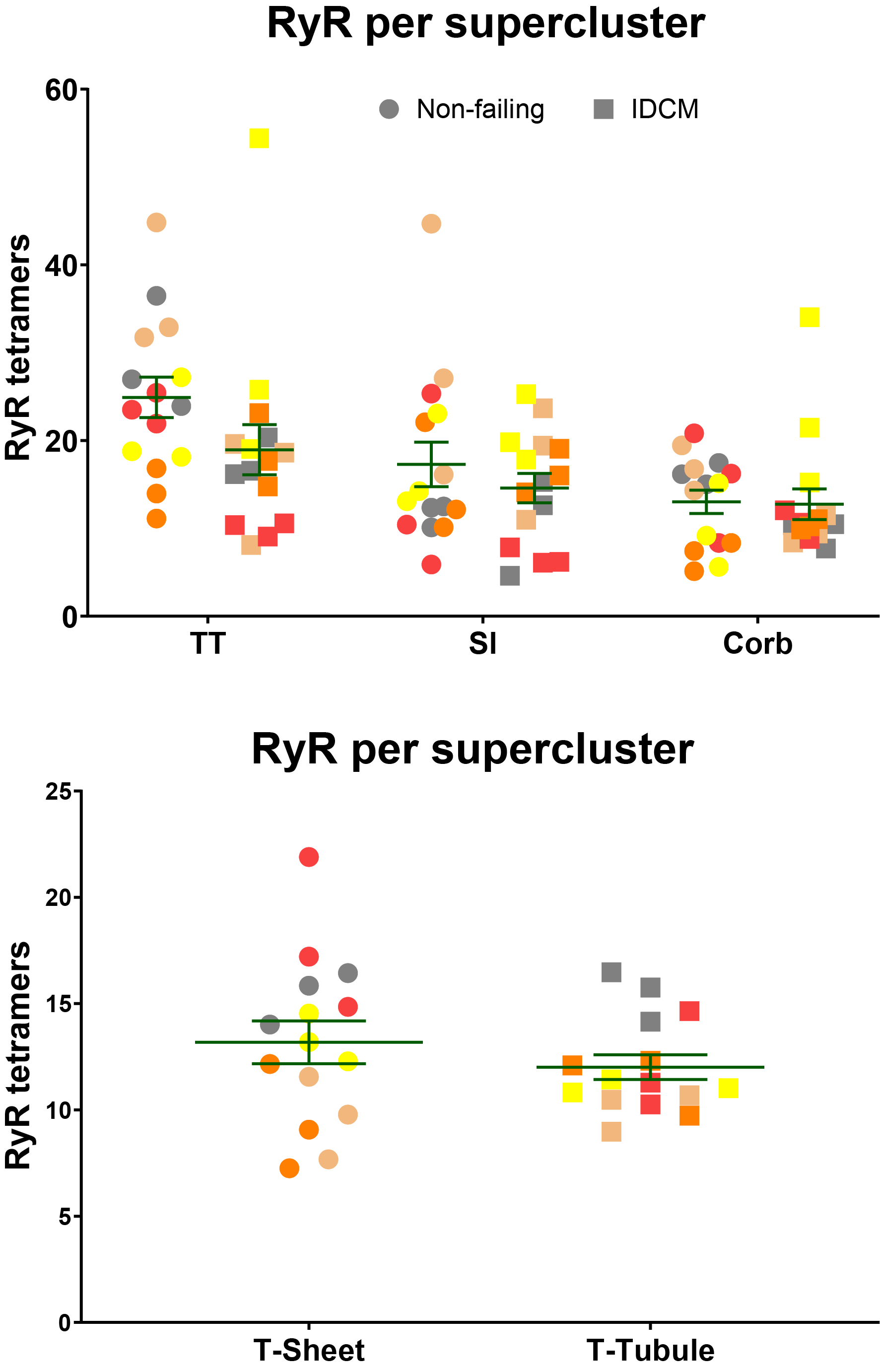


Sup Fig 2. RyR super cluster sizes. A) Mean RyR tetramers per supercluster for non-failing and failing myocytes at t-tubules (TT), sarcolemma (Sl), and corbular sarcoplasmic resticulum (Corb) in non-failing and failing cardiac myocytes. N=5 non-failing hearts and n=5 failing hearts with 3 cells analysed per heart. LME model was used to test for effects of disease status (IDCM, non-failing) and cell region (t-tubules, sarcolemma, corbular) and random effects were heart and cell (nested within heart). P values reported are post-hoc comparison of marginal means using Sidak test for multiple comparisons. (B) Mean RyR tetramers per supercluster for t-tubule and t-sheet regions. N=5 failing hearts with 3 cells examined for each region. **p<0.01, ***p<0.001. LME model was used to test for effects of region (t-sheets and t-tubules) and random effects were heart. For RyR super cluster size p=0.17.
